# Ultrasound Imaging of Brachial and Antebrachial Fasciae

**DOI:** 10.3390/diagnostics11122261

**Published:** 2021-12-02

**Authors:** Carmelo Pirri, Diego Guidolin, Caterina Fede, Veronica Macchi, Raffaele De Caro, Carla Stecco

**Affiliations:** Department of Neurosciences, Institute of Human Anatomy, University of Padua, 35121 Padua, Italy; diego.guidolin@unipd.it (D.G.); caterina.fede@unipd.it (C.F.); veronica.macchi@unipd.it (V.M.); rdecaro@unipd.it (R.D.C.); carla.stecco@unipd.it (C.S.)

**Keywords:** deep fascia, ultrasonography, arm, forearm, thickness, reliability

## Abstract

Knowledge about fasciae has become increasingly relevant in connection to regional anesthesiology, given the growing interest in fascial plane, interfascial, and nerve blocks. Ultrasound (US) imaging, thanks to high definition, provides the possibility to visualize and measure their thickness. The purpose of this study was to measure and compare, by US imaging, the thickness of deep/muscular fasciae in different points of the arm and forearm. An observational study has been performed using US imaging to measure brachial and antebrachial fasciae thickness at anterior and posterior regions, respectively, of the arm and forearm at different levels with a new protocol in a sample of 25 healthy volunteers. Results of fascial thickness revealed statistically significant differences (*p* < 0.0001) in the brachial fascia between the anterior and the posterior regions; in terms of the antebrachial fascia, no statistically significant difference was present (*p* > 0.05) between the regions/levels. Moreover, regarding the posterior region/levels, the brachial fascia had a greater thickness (mean 0.81 ± 0.20 mm) than the antebrachial fascia (mean 0.71 ± 0.20 mm); regarding the anterior region/levels, the antebrachial fascia was thicker (mean 0.70 ± 0.2 mm) than the brachial fascia (mean 0.61 ± 0.11 mm). In addition, the intra-rater reliability reported good reliability (ICC_2,k_: 0.88). US imaging helps to improve grading of fascial dysfunction or disease by revealing subclinical lesions, clinically invisible fascial changes, and one of the US parameters to reliably evaluate is the thickness in the different regions and levels.

## 1. Introduction

Knowledge about the fasciae has become increasingly relevant in connection to regional anaesthesiology, given the growing interest in fascial plane, interfascial, and nerve blocks. In fact, knowing the exact thickness of a patient’s fasciae reduces the risk of nerve damage during a peripheral block procedure and it makes it possible to predict the effect of compartment models of anesthetic drugs [1]. Several studies have also confirmed the importance of the deep/muscular fascia thickness in the acute compartment syndrome [2]. The latter is defined as a limb-threatening and occasionally life-threatening condition caused by bleeding or edema in a close muscle compartment surrounded by fascia or bone [3]. The body areas at risk of developing compartment syndrome are the limbs, and the most affected compartments are the anterior compartments in the leg and the forearm, due to their high fascial rigidity [3]. In both, the main treatment is fasciotomy [2]. So, in recent years numerous investigations have been carried out on this topic to understand the anatomical features of the various fasciae of the body [2,4], both in cadavers and in live individuals using Ultrasound (US) Imaging [5,6,7,8]. The latter, thanks to its high definition, the possibility to visualize the musculo–skeletal structures in a dynamic way and the lower cost when compared to other non-invasive methods [8,9,10], it has become an important tool for studying fascial anatomy and pathology in a rehabilitative point of view [5]. Indeed, with US, it is possible to demonstrate the thickening of a fascial layer, the changing of its echogenicity, and to analyze the relationships between fasciae, nerves, and vessels. According to some studies [11,12], it is also possible to investigate the gliding between muscles and the adjacent fascial layers, and between the various fascial layers, that seem to be related to myofascial pain. A review by Fede et al. [13] reported that the ultrasound data collected for the same fascia differed depending on the ultrasonographer, probe position, and/or intra-individual anatomic variability. Therefore, it is mandatory, beforehand, to speak about fascial alteration in pathological conditions, to have a clear idea about the normal aspect of each fascia of the body, and to codify the best probe position to visualize them. While this knowledge is present for the thoracolumbar fascia and the fasciae of the inferior limbs, to date, no study has evaluated the fascial thicknesses of the brachial and antebrachial fasciae measured by ultrasound imaging in the different points. For the antebrachial fascia, it is only known that high-definition US imaging allows its identification in relationships with the adjacent structures [2,14,15].

Furthermore, it is well known from dissection that in the upper limb, the deep fasciae have aponeurotic features, being a strong, almost white laminar sheet of connective tissue that covers the muscles. Collagen fiber bundles arranged in different directions are easily identifiable within this fascia [16,17].

The main purpose of this study was to codify the best positions for the probe to study the deep fasciae of the upper limb and to understand if they have constant features in the various areas, or if they present different thicknesses in different regions and levels of the arm and of the forearm. The second aim was to assess the intra-rater reliability of the US evaluation of the deep fasciae of the upper limb.

## 2. Materials and Methods

### 2.1. Study Design

A cross-sectional study based on the Strengthening the Reporting of Observational studies in Epidemiology (STROBE) statement was conducted [18] in order to compare the US thicknesses of brachial and antebrachial fasciae in different compartment and levels of the arm and of the forearm. The Helsinki Declaration and human experimentation rules [19] were considered and the Ethics Committee of University of Padua evaluated the research. All participants were informed prior to inclusion in the project by providing a written consent form.

### 2.2. Participants

A total sample of 25 subjects were recruited aged between 20 and 60 years. The participants were excluded if they had any upper extremity injuries (e.g., previous fractures, tendinopathies, tendon ruptures, or neuropathy injuries; past diagnosis of a neuromusculoskeletal condition of the arm or forearm, e.g., use of palmar orthoses, carpal tunnel syndrome, etc.; or past diagnosis of a neuromusculoskeletal condition of the arm and forearm, e.g., degeneration or inflammation of the homerus periosteum) or surgery, severe orthopedic, neuronal, psychiatric, cardiopulmonary, or endocrine diseases, were under 18 years old, pregnant, with a chronic skin condition (eczema, psoriasis, etc.), had previous severe trauma in the inferior limbs, collagen disorder (scleroderma, mixed connective tissue disorder, etc.), and/or chronic medical condition requiring intake of medications. The enrolment of the subjects was performed by a specialized medical doctor with more than 5 years of experience in physical and rehabilitation medicine.

### 2.3. Ultrasonography Imaging Measurements

Using a high-resolution device (Sonosite Edge II, FUJIFILM, Inc. 21919, Bothell, WA, USA) with a 6–15 MHz linear transducer (HFL50x, Sonosite Edge II, FUJIFILM, Inc. 21919, WA, USA) and a screen resolution of 1680 × 1050 pixels, ultrasound images were taken of the arm and forearm with a specific US scans protocol. A physician specialist in Physical and Rehabilitation Medicine with 7 years’ experience in skeletal-muscle US imaging and US imaging of fasciae carried out the US measurements.

A standardized protocol was created and used to assess the fascial layers (deep fascia/brachial fascia and deep fascia/antebrachial fascia in the different compartments and levels for bilateral assessment.

The US system speed of sound was c = 1540 m/s, conventionally used in diagnostic US system. The US was set to B-mode and depicted a depth of 30 mm. For adequate scans and to reduce surface pressure on the skin, the ultra-sonographer used suitable amounts of gel. The probe was placed on the skin as lightly as possible to avoid tissue compression but quite stable to maintain adequate contact between the probe and skin for consistent images. The US beam was kept perpendicular to the fascial layers because anisotropy artifacts typically affect them. The power and overall gain of the ultrasound machine were adjusted to optimize visualization of the fascial planes and obtain the best scans possible [20]. The investigator used the short axis because this is the best method to visualize and follow the landmarks correlated with the fascial layers and to have less spatial anisotropy [21].

The US images were frozen, captured and acquired at the end of each assessment; the fascial thickness was measured using Image J software. To eliminate the influence of possible thickness variations, three equidistant regions of interest per image/level for fascia were measured; in each of them, three points representing the best visibility for each fascial layer were measured and the resulting values were averaged for analysis. The rater followed the same protocol to ensure that each point of brachial and antebrachial fasciae of the arm and forearm were quantified in the same way. Moreover, the same procedure of image assessment was repeated three different times to calculate the reliability of the measurements.

For each point, following the description of the fascial layers visualization in ultrasound imaging used by Pirri et al. [6], a specific protocol was defined:

#### 2.3.1. Arm

**Figure 1:**
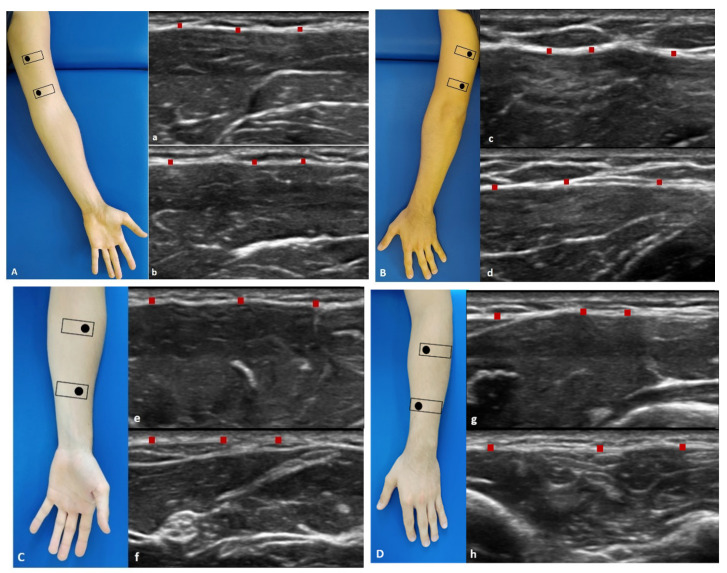
Ultrasound (US) images of: the anterior region of the arm (**A**) and of the forearm (**C**); the posterior region of the arm (**B**) and of the forearm (**D**). Anterior region (**A**,**C**) at the levels Ant 1 (**a**,**e**) and Ant 2 (**b**,**f**). Posterior region (**B**,**D**) at levels Post 1 (**c**,**g**) and Post 2 (**d**,**h**). Probe: black rectangle. Red dashes: deep fascia.

1. Anterior region (Figure 1A): the patient was in a relaxed supine position with the upper limb in a neutral position.
  Anterior 1: the probe is placed axially, over the proximal half of the anterior arm. The median nerve and brachial artery lie medially between the brachialis and triceps muscles (Figure 1(Aa)).
  Anterior 2: the probe was axially moved downwards following the median nerve and brachial artery. The brachialis muscle can be seen deep to the biceps muscle (Figure 1(Ab)).
2. Posterior region (Figure 1B): the patient was prone with the upper limb in a neutral position.
  Posterior 1: the probe is placed axially, over the proximal half of the posterior arm. Each belly of the triceps muscle can be seen separately. The median nerve, ulnar, and radial nerves can be seen in close proximity. The brachial artery and vein should be taken into consideration with the nerves as landmarks (Figure 1(Bc)).
  Posterior 2: the probe was axially moved downwards following the landmarks (Figure 1(Bd)).

#### 2.3.2. Forearm

1. Anterior region (Figure 1C): the patient was in a relaxed supine position with the upper limb in a neutral position and the forearm in supination position.
  Anterior 1: the probe was axially placed on the anterior proximal half of the forearm. The pronator teres lies lateral to the flexor carpi radialis (FCR) and flexor digitorum superficialis (FDS). The median nerve can be seen between the two heads of the pronator teres muscle (Figure 1(Ce)).
  Anterior 2: the probe was axially moved downwards, over the distal half of the anterior forearm. Flexor digitorum profundus (FDP) is seen deep in the FDS, flexor carpi ulnaris (FCU), and palmaris longus. These structures lay over the interosseus membrane (Figure 1(Cf)).
2. Posterior region (Figure 1D): the patient was prone with the upper limb in a neutral position and the forearm is in pronation position.
  Posterior 1: place the probe axially over the dorsal aspect of the proximal half of the forearm. At this level extensor digitorum (ED) lies between extensor carpi radialis brevis (ECRB) and extensor digiti minimi (EDM) over the supinator muscle. The brachioradialis muscle can be observed lying medial to the extensor carpi radialis longus (ECRL) and brevis (ECRB) muscles. Adjacent branches of the radial nerve and artery can be seen (Figure 1(Dg)).
  Posterior 2: the probe was axially moved downwards, over the distal dorsal half of the forearm. ED becomes smaller, while EDM becomes larger. The abductor pollicis longus (APL) lies adjacent to radius bone, and the extensor pollicis longus (EPL) lies adjacent to ulna (U). The posterior interosseus nerve is located between the extensor digitorum superficially. The extensor carpi ulnaris (ECU) is the most medially located extensor muscle lying over the ulna (U), medial to the extensor digiti minimi (EDM) and EPL and APL deeply. The extensor pollicis brevis (EPB) and the extensor indicis (EI) can already be seen (Figure 1(Dh)).

### 2.4. Statistical Analysis

Statistical analysis was performed using GraphPad PRISM 8.4.2 (GraphPad Software Inc., San Diego, CA, USA), and a *p* < 0.05 was always considered as the limit for statistical significance. The resulting effect size was calculated by G Power 3.1 (Universität Düsseldorf: Psychologie) according to Cohen’s d and interpreted as small (d = 0.20), medium (d = 0.50), and large (d = 0.80) [22]. For brachial and antebrachial fasciae, the effect size was d = 1 in a first our pilot study confirmed from other study [11], α error prob = 0.05, power: 1-β err prob = 0.95; total sample size was = 13 [22]. Nevertheless, we could include a sample of 25 individuals in our group.

The normality assessment was carried out using the Kolgomorov–Smirnov test. Descriptive statistics were calculated, including measures of central tendency and their dispersion ranges using mean and standard deviation (SD) to describe parametric data. Differences in US-estimated thickness of the brachial fascia and antebrachial fascia across regions/levels were statistically analyzed by one-way analysis of variance (ANOVA) followed by Tukey’s test for multiple comparisons. In addition, the Pearson’s test was employed for both groups to evaluate the correlation between BMI, weight, height, age, and brachial fascia or antebrachial fascia.

Moreover, two-way mixed model intra-class correlation coefficient (ICC 2, k), type A, k, was used to evaluate the intra-rater reliability. ICC values were interpreted as poor when below 0.5, as moderate when between 0.5 and 0.75, as good when between 0.75 and 00.90, and as excellent when above 0.90 [23]. SPSS version 21 was used for this analysis of reliability (SPSS Inc., Chicago, IL, USA).

## 3. Results

A total of 25 subjects (13 female and 12 male) participated in this study. The descriptive data of the sample were summarized in Table 1.

### 3.1. Ultrasound Measurements of the Brachial Fascia (Deep Fascia of the Arm)

**Figure 2:**
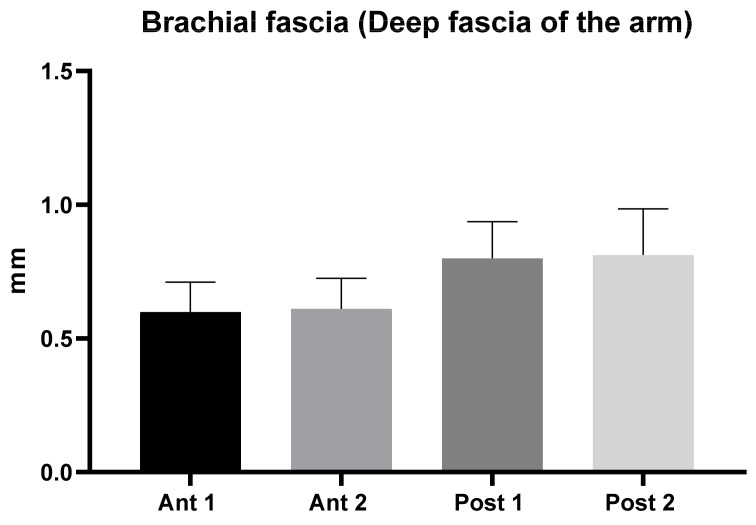
Ultrasound thickness measurements of the Brachial fascia of the arm.

The brachial fascia had a mean US thickness of 0.71 ± 0.13 mm (Table 2 and Figure 2).

The brachial fascia was thicker (*p* < 0.0001) in the posterior region (0.81 ± 0.2 mm) respect to the anterior region (0.61 ± 0.11 mm); whilst there was no difference between the proximal and the distal levels (Table 3). In addition, the comparison within different regions/levels of the brachial fascia are reported in Table 3. According to Tukey’s multiple comparisons test, the comparison between brachial fascia thickness among various levels/regions of the arm showed a statistically significant difference (Table 3).

### 3.2. Ultrasound Measurements of the Antebrachial Fascia (Deep Fascia of the Forearm)

**Figure 3:**
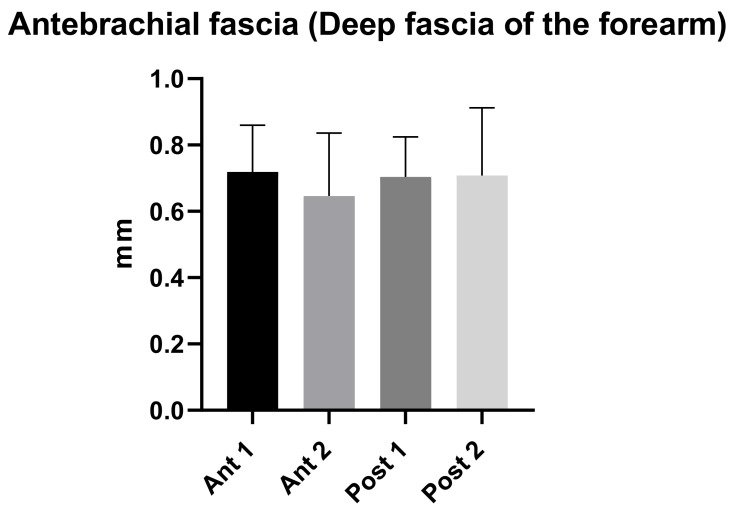
Ultrasound thickness measurements of the Antebrachial fascia of the forearm.

The antebrachial fascia had a mean US thickness of 0.70 ± 0.2 mm (Table 4 and Figure 3).

The antebrachial fascia had a mean thickness of 0.71 ± 0.2 mm in the posterior region compared to a mean thickness of 0.68 ± 0.2 mm in the anterior region (Table 4). In addition, the comparison within different regions/levels of the antebrachial fascia are reported in Table 5. According to Tukey’s multiple comparisons test, the comparison between antebrachial fascia thickness among various levels/regions of the forearm did not show a statistically significant difference (*p* > 0.05) (Table 5).

### 3.3. Ultrasound Measurements Comparison between the Brachial Fascia and the Antebrachial Fascia

**Figure 4:**
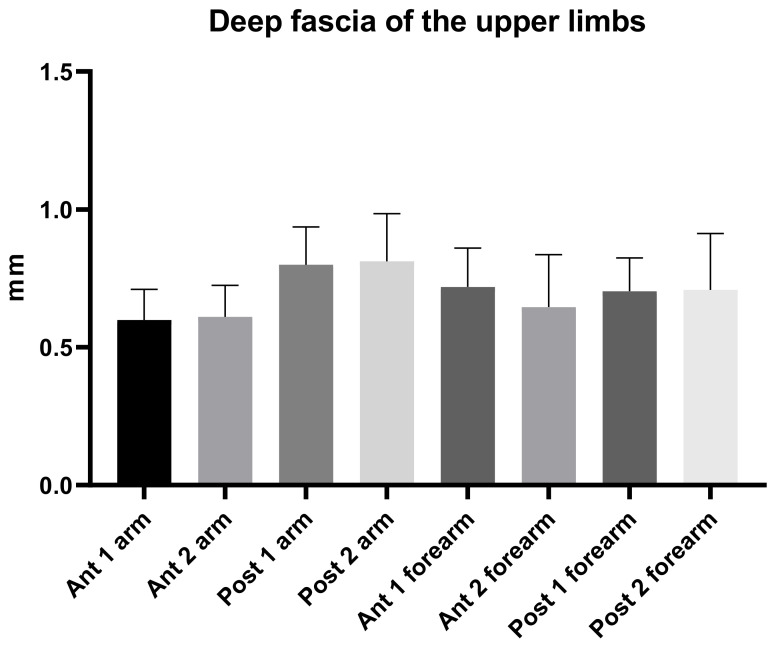
Ultrasound thickness measurements of the Brachial and Antebrachial fascia at different regions/levels.

According to Tukey’s multiple comparisons test (Table 6 and Figure 4), the comparison between different regions/levels of the brachial and the antebrachial fascia showed a statistically significant difference with an alternating trend between the anterior and posterior region of the brachial and antebrachial fasciae (Table 6). Regarding the posterior region/levels, the brachial fascia had a greater thickness (mean 0.81 ± 0.20 mm) than the antebrachial fascia (mean 0.71 ± 0.20 mm) (Figure 4 and Table 6); in terms of the anterior region/levels, the antebrachial fascia was thicker (mean 0.70 ± 0.2 mm) than the brachial fascia (mean 0.61 ± 0.11 mm).

### 3.4. Intra-Rater Reliability

In addition, the intra-rater reliability was reported as good. The results for brachial fascia were: anterior region (ICC_2,k_: 0.88; 0.85–0.90), and posterior region (ICC_2,k_:0.88; 0.85 –0.90). Antebrachial fascia: anterior region (ICC_2,k_: 0.89; 0.85–0.92), and posterior region (ICC_2,k_: 0.88; 0.85–0.90) (Table 7).

## 4. Discussion

To the current knowledge, this study may be stated as the first study detailing the brachial fascia and the antebrachial fascia US thicknesses at the different regions and levels.

As has been reported by other studies examining the deep fascia in other topographical regions (thigh and leg) by US imaging [7,8], the brachial and antebrachial fasciae were visualized in all regions and levels, appearing as linear hyperechogenic layers, below the subcutaneous tissue that surround the muscles [6].

The study’s primary aim was to investigate the difference of the fascia thickness of the brachial and antebrachial fascia in different regions and levels of the arm and of the forearm among healthy volunteers.

An analysis of our results about brachial fascia showed that in the posterior region at the different levels was thicker (0.81 ± 0.20 mm) than the anterior region (0.61 ± 0.11 mm) (Table 2), showing a statistical difference (*p* < 0.0001) (Table 3 and Figure 2), similar to the trend that is present in the lower limbs [7,8]. On the contrary, the antebrachial fascia showed no statistically significant difference between the anterior and the posterior regions/levels (Table 5 and Figure 3). Its US thickness was of 0.70 ± 0.20 mm for the anterior region and of 0.71 ± 0.20 mm for the posterior region (Table 4). In addition, the brachial fascia was always thicker in the posterior region/levels (mean 0.81 ± 0.20 mm) than the posterior region/levels of the antebrachial fascia (mean 0.71 ± 0.20 mm) (Figure 4 and Table 6); it was also thicker proximally, probably for the presence at this level of the myofascial expansion of the latissimus dorsi muscle. In the same way, the presence of the myofascial expansion of the lacertus fibrosus can also explain why the Ant 1 of the antebrachial fascia was thicker (0.72 ± 0.14 mm) than the Ant 1 of the brachial fascia (0.60 ± 0.11 mm).

In the light of these findings, the deep fasciae of the arm and forearm, which tend to be thicker posteriorly, play the important role of myofascial force transmission in these compartments [24]. They are also thicker in the proximal levels, compatibly with the role of the attachment and myofascial expansion of the arm and forearm muscles [25,26]. Indeed, the brachial and antebrachial fasciae form a unique sheath that might be compared to an evening glove, proximally tensioned by the various myofascial insertions of the pectoral girdle muscles. This glove is partially free to glide over the underlying muscular plane, but at some points it attaches to bones or inserts into muscular fibers. Contraction of these muscular fibers stretches the deep fasciae in specific directions [24]. All the muscles of the pectoral girdle send myofascial expansions to the brachial fascia (CTO), and, consequently, the brachial fascia is thicker respect to the antebrachial fascia.

The results confirmed, as has been demonstrated by other studies, that there are a good intra- and inter-reliability in the US assessment of the deep fasciae, in the case of sonographers with optimal US technical skills and fascial anatomy knowledge [8,27,28].

Based on the evidence of the increasingly relevant deep fasciae role in connection to regional anaesthesiology, and given the growing interest in fascial plane, inter-fascial and nerve blocks, it can be reported that the exact thickness of a patient’s arm and forearm fasciae reduces the risk of nerve damage during these procedures [1]. Furthermore, understanding the thickness of the brachial and antebrachial fasciae is crucial for the fascial plane blocks, influencing the deposit and spread of local anesthetic, not only in the effectiveness of the blocks (i.e., onset time and area of anesthesia) [29], but also in the postoperative analgesia [30].

Moreover, the importance of the deep/muscular fascia thickness in the acute and chronic compartment syndrome suggests the use of US imaging to evaluate fascial thickness in the case of these pathologies as an inexpensive, safe, non-invasive, portable, and most of all, effective tool [2].

This is the first work to our knowledge to examine and compare the thicknesses of the deep fascial layers of the arm and forearm using US imaging. Future longitudinal studies including not only healthy volunteers, but also large numbers of patients will be able to contribute to our knowledge of the pathophysiology of different thickness patterns. US may also be able to uncover changes which are invisible during clinical inspection and unforeseen by current clinical practice. Finally, being able to define the specific structures involved in fascial dysfunctions would facilitate a more targeted approach to treatment.

### Limitations of the Study

The small number of healthy volunteers included in this study cohort and the qualitative aspect of the assessments mean that it is not possible to statistically analyze the prevalence of US findings as well as to explain their possible causes, prognostic significance, and therapeutic implications.

Finally, US evaluation of fascia morphology greatly depends on the skill of the investigator as well as proper setting of the device.

## 5. Conclusions

US refines visual evaluation of the fascial layers in patients with various musculo–skeletal problems, being an inexpensive, safe, non-invasive, portable, and, most of all, effective instrument that can help clinicians to better understand fascial pathology. In addition, it may reveal changes not highlighted by the normal clinical inspection. A few of these changes require further investigations because they have not yet been explained or described. Accordingly, US may help to improve grading of fascial dysfunction or disease by revealing subclinical lesions and clinically invisible fascial changes, and one of the US parameters to evaluate is the thickness in the different regions and levels.

## Figures and Tables

**Table 1:** Descriptive data of the sample.

Descriptive Statistics	Age	BMI	Height	Weight
Number of values	25	25	25	25
Minimum	20	15.79	158	43
Maximum	60	31.6	183	87
Range	40	15.81	25	44
Mean	32.72	23.61	171.1	69.5
Std. Deviation	13.48	3.594	7.224	13.06
Coefficient of variation	41.19%	15.23%	4.222%	18.79%

**Table 2:** Ultrasound thickness measurements of the Brachial fascia of the arm.

Descriptive Statistics	Ant 1	Ant 2	Post 1	Post 2
Number of values	50	50	50	50
Minimum	0.43	0.4	0.47	0.5
Maximum	1.11	0.9	1.2	1.26
Range	0.68	0.5	0.73	0.76
Mean	0.60	0.61	0.80	0.81
Std. Deviation	0.11	0.11	0.14	0.20
Std. Error of Mean	0.02	0.02	0.02	0.02
Coefficient of variation	18.62%	18.72%	17.16%	21.27%

**Table 3:** Ultrasound measurements comparison within different regions/levels of the brachial fascia. Statistically significant results are showed in bold. ****: *p* < 0.0001. Ns: not statistically significant.

Type of Comparison	Mean Diff.	95.00% CI of Diff.	Significant?	Summary	Adjusted *p* Value
Ant 1 vs. Ant 2	−0.0114	−0.08196 to 0.05916	No	ns	0.9752
Ant 1 vs. Post 1	−0.2006	−0.2712 to −0.1300	**Yes**	****	**<** **0.0001**
Ant 1 vs. Post 2	−0.213	−0.2836 to −0.1424	**Yes**	****	**<0.0001**
Ant 2 vs. Post 1	−0.1892	−0.2598 to −0.1186	**Yes**	****	**<0.0001**
Ant 2 vs. Post 2	−0.2016	−0.2722 to −0.1310	**Yes**	****	**<0.0001**
Post 1 vs. Post 2	−0.0124	−0.08296 to 0.05816	No	ns	0.9685

**Table 4:** Ultrasound thickness measurements of the Antebrachial fascia of the forearm.

Descriptive Statistics	Ant 1	Ant 2	Post 1	Post 2
Number of values	50	50	50	50
Minimum	0.44	0.34	0.49	0.51
Maximum	1.1	1.04	1.1	1.1
Range	0.66	0.7	0.61	1.049
Mean	0.72	0.70	0.70	0.71
Std. Deviation	0.14	0.20	0.12	0.20
Std. Error of Mean	0.02	0.03	0.02	0.03
Coefficient of variation	19.58%	29.35%	17.19%	28.81%

**Table 5:** Ultrasound measurements comparison within different regions/levels of the antebrachial fascia. Ns: not statistically significant.

Type of Comparison	Mean Diff.	95.00% CI of Diff.	Significant?	Summary	Adjusted *p* Value
Ant 1 vs. Ant 2	0.0726	−0.01414 to 0.1593	No	ns	0.1356
Ant 1 vs. Post 1	0.0156	−0.07114 to 0.1023	No	ns	0.9664
Ant 1 vs. Post 2	0.01078	−0.07596 to 0.09752	No	ns	0.9884
Ant 2 vs. Post1	−0.057	−0.1437 to 0.02974	No	ns	0.325
Ant 2 vs. Post 2	−0.06182	−0.1486 to 0.02492	No	ns	0.2547
Post 1 vs. Post 2	−0.00482	−0.09156 to 0.08192	No	ns	0.9989

**Table 6:** Ultrasound measurements comparison between different regions/levels of the brachial and the antebrachial fascia. Statistically significant results are showed in bold. *: *p* < 0.05; **: *p* < 0.01; ****: *p* < 0.0001. Ns: not statistically significant.

Type of Comparison	Mean Diff.	95.00% CI of Diff.	Significant?	Summary	Adjusted *p* Value
**Ant 1 arm vs. Ant 1 forearm**	−0.12	−0.213 to −0.027	**Yes**	**	**0.0025**
Ant 1 arm vs. Ant 2 forearm	−0.05	−0.140 to 0.045	No	ns	0.7776
**Ant 1 arm vs. Post 1 forearm**	−0.11	−0.197 to −0.011	**Yes**	*	**0.0157**
**Ant 1 arm vs. Post 2 forearm**	−0.11	−0.202 to −0.016	**Yes**	**	**0.0092**
**Ant 2 arm vs. Ant 1 forearm**	−0.11	−0.201 to −0.015	**Yes**	**	**0.0098**
Ant 2 arm vs. Ant 2 forearm	−0.04	−0.129 to 0.056	No	ns	0.9373
**Ant 2 arm vs. Post 1 forearm**	−0.09	−0.186 to −1.345	**Yes**	*	**0.0499**
**Ant 2 arm vs. Post 2 forearm**	−0.10	−0.191 to −0.005	**Yes**	*	**0.0312**
Post 1 arm vs. Ant 1 forearm	0.08	−0.012 to 0.174	No	ns	0.1444
**Post 1 arm vs. Ant 2 forearm**	0.15	0.060 to 0.246	**Yes**	****	**<0.0001**
**Post 1 arm vs. Post 1 forearm**	0.10	0.003 to 0.20	**Yes**	*	**0.0367**
Post 1 arm vs. Post 2 forearm	0.09	−0.001 to 0.184	No	ns	0.0581
**Post 2 arm vs. Ant 1 forearm**	0.09	1.345 to 0.20	**Yes**	*	**0.0499**
**Post 2 arm vs. Ant 2 forearm**	0.17	0.072 to 0.260	**Yes**	****	**<0.0001**
**Post 2 arm vs. Post1 forearm**	0.11	0.020 to 0.205	**Yes**	**	**0.0098**
**Post 2 arm vs. Post 2 forearm**	0.10	0.011 to 0.20	**Yes**	*	**0.0168**

**Table 7:** Intra-rater reliability of the ultrasound measurements within different regions/levels of the brachial and of the antebrachial fascia.

Type of Fascia	Region	ICC
Brachial fascia	Anterior	0.88 (0.85–0.90)
Brachial fascia	Posterior	0.88 (0.85–0.90)
Antebrachial fascia	Anterior	0.89 (0.85–0.92)
Antebrachial fascia	Posterior	0.88 (0.85–0.90)

## Data Availability

The data presented in this study are available on request from the corresponding author. The data are not publicly available due to privacy.
